# Additional Observations on Phos. of Lime in Depraved States

**Published:** 1852-03

**Authors:** 


*Additional Observations on Phosphate of Lime in Depraved States.* By WARREN STONE, M. D., Prof. of Surgery in Med. Dep. Un'ty of La.

The brief article published in the first number of the Register, on the use of the phosphate of lime in phthisis, and other depraved states of the system, has been published in some of the newspapers of the country, and I deem it proper to make some further remarks, lest disappointment should follow its indiscriminate use. The best way to secure for any remedy its proper place in therapeutics, is to determine its mode of action. It is considered, by the best observers, that in scrofulous affections there is undue acidity of the stomach; and, in the opinion of Dr. Thompson, the alkali of the salivary and pancreatic fluids being neutralized, fails to convert the carbon into oil. The blood is overcharged with albumen, and the albuminous exhalations being deficient in fat, elementary molecules are not formed capable of development into cells, and tubercular corpuscles are the result. Whether this theory is correct or not, I have always found the good effects of the lime manifest where this acid tendency was manifest, and it has always appeared sufficient to correct it. My experience is, that the cod liver oil is much better tolerated by the stomach when taken with the phosphate of lime; and I feel confident that it is better appropriated. It is well understood that cod liver oil, to be useful, must be digested, and furnish to the blood certain essential principles known to be deficient in phthisical cases. The phosphate of lime undoubtedly corrects the acidity, and experience goes strongly to favor the theory of Beneke, that it assists in the formation of healthy nuclei, capable of development into cells. When the oil is not tolerated, great benefit is derived from the use of the lime in connection with nitrogenous diet, or animal oil, in the form of diet. Several cases have been reported to me, where the good effects of the cod liver oil were not manifest until the lime was added. In urging strongly the use of the lime in connection with cod liver oil and animal diet, or animal oil, I do not wish to be understood as undervaluing other agents, which the various conditions of the fluids and nervous system often require. In this section, and in the whole valley of the Mississippi, there is a tendency to intermittents, engorgement of the spleen, and consequent deficiency of coloring matter in the blood, in which the preparations of iron are highly useful. The carbonate of iron, prussiate of iron, iodide of iron, and in decidedly intermittent cases, the citrate of quinine and iron, are highly useful. Exercise, particularly such as is calculated to increase the capacity of the chest, and favor free decarbonization of the blood, should not be overlooked. The chief difficulty in private practice, in the use of the main remedies in phthisis, is in the want of confidence, and consequently, perseverance in their use. The patient derives temporary relief from some one of the thousand quack specifics, which merely disguise symptoms, but have no curative virtues. But few can comprehend that a transformation of tissues, dependent upon vice of nutrition, can only be overcome by long perseverance in a course calculated to correct it. Cod liver oil was used — and with a confidence equal to any modern physician — seventy-five years ago; but it went into disuse, probably from ignorance of its mode of action, and consequently, want of confidence in its use. The effect of the phosphate of lime in aid of the proper appropriation of the nutriment, is now manifest in certain cases of marasmus, not dependent upon organic disease, but equally destructive. The food, at times, appears to be digested, and, by the use of gentle means, stayed upon the bowels; but nutrition does not go on; there is no appropriation of food. In such cases, I have seen the lime, in conjunction with animal juices, and even with animal fat, produce the most happy effects. I will not pretend that the theory of the action of the lime is entirely correct, but I am sure I am not mistaken in its effects in favoring the healthy appropriation of nutriment, and even in favoring digestion. — *New Orleans Monthly Register.*

